# A Two-Stage Random Forest-Based Pathway Analysis Method

**DOI:** 10.1371/journal.pone.0036662

**Published:** 2012-05-07

**Authors:** Ren-Hua Chung, Ying-Erh Chen

**Affiliations:** 1 Division of Biostatistics and Bioinformatics, Institute of Population Health Sciences, National Health Research Institutes, Zhunan, Miaoli, Taiwan; 2 Center for Genetic Epidemiology and Statistical Genetics, John P. Hussman Institute for Human Genomics, University of Miami Miller School of Medicine, Miami, Florida, United States of America; 3 Department of Economics, North Carolina State University, Raleigh, North Carolina, United States of America; Institute of Psychology, Chinese Academy of Sciences, China

## Abstract

Pathway analysis provides a powerful approach for identifying the joint effect of genes grouped into biologically-based pathways on disease. Pathway analysis is also an attractive approach for a secondary analysis of genome-wide association study (GWAS) data that may still yield new results from these valuable datasets. Most of the current pathway analysis methods focused on testing the cumulative main effects of genes in a pathway. However, for complex diseases, gene-gene interactions are expected to play a critical role in disease etiology. We extended a random forest-based method for pathway analysis by incorporating a two-stage design. We used simulations to verify that the proposed method has the correct type I error rates. We also used simulations to show that the method is more powerful than the original random forest-based pathway approach and the set-based test implemented in PLINK in the presence of gene-gene interactions. Finally, we applied the method to a breast cancer GWAS dataset and a lung cancer GWAS dataset and interesting pathways were identified that have implications for breast and lung cancers.

## Introduction

Many genome-wide association studies (GWAS) have been conducted to identify markers associated with diseases over millions of SNPs. However, to survive the multiple testing correction over millions of tests, SNPs need to have strong marginal effects on the disease or a large sample size is required for SNPs with small effects. For a complex disease that is often caused by the joint effects of multiple genes with small marginal effects, considering the effects jointly will significantly increase the statistical power to identify these genes. Pathway analysis provides a powerful approach for identifying the joint effect of genes grouped into biologically-based pathways on disease. Promising pathway results have already been identified in GWAS datasets [1]–[3].

Recently, many statistical pathway analysis methods have been proposed. Most of them focused on testing the cumulative main effects of genes in a pathway [4]–[6]. That is, pathway statistics were derived based on single-marker association test statistics or p-values. However, for complex diseases, gene-gene interactions are expected to play a critical role in disease etiology. Some methods, such as “Gene set Ridge regression in Association studies” (GRASS) [6], which is based on a regression framework, can incorporate gene-gene interactions in the test. However, since there are many combinations of SNPs for interactions, it is not straightforward to select the combinations of SNPs in the regression model to account for gene-gene interactions.

Random Forest (RF) has been used as a tool for association tests [7], [8]. SNPs are used as predictor variables and disease status is the outcome in a classification tree. A set of classification trees is created based on replicates of samples generated by a bootstrap approach in the RF algorithm. The significance of a SNP is then evaluated based on its prediction ability for the disease outcome. Moreover, interactions are implicitly modeled in RF as each path of edges in the tree corresponds to a particular combination of alleles that is associated with the disease status [9]. Therefore, several studies also applied RF to test gene-gene interactions [10], [11]. RF is efficient for a gene-gene interaction analysis, since a small set of SNPs is used in each node of the tree for splitting the samples.

RF has also been shown to be useful for pathway analysis due to its promising feature of considering both main effects and gene-gene interactions. Pang et al. identified candidate pathways by ranking the pathways using their prediction error rates calculated in RF for gene expression data [12]. Chang et al. performed a pilot study of applying RF to SNP data for identifying pathways associated with Glioma [13]. A permutation procedure was used to estimate the p-value for each pathway by testing the significance of the prediction error rate calculated based on a set of SNPs within the pathway with respect to the error rates observed by chance. Although RF was demonstrated to be a useful approach for pathway analysis of SNP data in Chang et al., its statistical power for analyzing SNP data has not been evaluated by simulation studies. Moreover, a large pathway can have hundreds of genes, which can include thousands of markers. To test a large pathway for association, using all SNPs in the pathway for the RF pathway analysis may reduce the classification power, as more noise is introduced to the model. Reducing the number of SNPs that are not associated with the disease without biasing the results can significantly increase the power for RF.

Here we propose a powerful two-stage RF-based pathway test (TRF-pathway) based on SNP data, such as data from a GWAS. We used simulations to verify that the TRF-pathway has the correct type I error rates. We also compared the power of the TRF-pathway to the original RF-based pathway test used in Chang et al. Finally, we applied the TRF-pathway to a breast cancer GWAS dataset and a lung cancer GWAS dataset and the TRF-pathway identified candidate pathways that have implications for breast and lung cancer etiology.

## Methods

In the RF algorithm [14], a training set of samples are selected by sampling with replacement from the original samples. The training set is used to create a classification tree, and the remaining samples that are not in the training set are used as the testing set for the classification tree. The process is repeated a large number of times so that a forest of classification trees is created. Based on the forest of trees, a sample that is classified more often in a category (when it is in the testing set) is assigned to the category. A classification error rate can then be calculated based on the number of samples that are incorrectly classified. Moreover, the significance for each predictor variable can be assessed by a permutation procedure in RF. The variable importance is standardized to a *Z* score.

We incorporated the RF algorithm in the TRF-pathway test. SNPs in genes in a pathway are used as predictor variables to classify the case and control status in RF. For a large pathway, using SNPs in all the genes in the pathway may reduce the power for classification, because a majority of SNPs may not have effects on the disease. Therefore, we used a two-stage approach to eliminate the number of SNPs that may not have effects. The RF algorithm is performed on all SNPs in a pathway at the first stage. Then SNPs with variable importance scores greater than a user-specified threshold are selected at the second stage and the RF algorithm is performed only on the significant SNPs. The algorithm for the TRF-pathway is described as follows:

### The TRF-pathway algorithm

For each pathway, we perform the following steps in the TRF-pathway algorithm:

1. Select a set of SNPs within a user-specified distance to genes in a pathway.
2. RF is performed based on the set of SNPs and the standardized variable importance score is calculated for each SNP.
3. SNPs with importance scores greater than a user-specified threshold are selected as the important SNPs. RF is performed again based on the important SNPs.
4. The prediction error rate, which is the proportion of samples not correctly categorized, from the RF analysis in step 3 is used as a score *R* for the pathway.
5. Permute the case-control affection status and repeat steps 2–4 *K* times. In each permutation *i*, the score *R_i_* is calculated.
6. The p-value for the pathway is calculated as: 
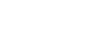
, where *I*(*S*) = 1 when the statement *S* is true and *I*(*S*) = 0 when *S* is false. The null hypothesis for the TRF-pathway is that none of the SNPs in the pathway are associated with the disease.

Note that if the user does not specify a threshold for the variable importance scores, all the SNPs within genes in a pathway are used for RF analysis and the TRF-pathway algorithm is reduced to the RF-based pathway algorithm used in Chang et al. [13]. In the following text, we refer to the RF-pathway as the method used in Chang et al.

Unlike methods developed for GWAS data that compare test statistics of genes in a pathway with respect to statistics for background genes in the genome such as Wang's method [1] and “Association LIst GO Annotator” (ALIGATOR) [5], the TRF-pathway compares the test statistic (i.e. the prediction error) with respect to the null distribution of the test statistic. Therefore, the TRF-pathway is suitable not only for GWAS, but also for candidate gene or candidate pathway studies.

### Simulations

We conducted simulation studies to evaluate the type I error rates and power for the TRF-pathway. We used genomeSIMLA to simulate SNPs in genes in a pathway based on Affymetrix 550k chip [15]. Linkage Disequilibrium (LD) structures for SNPs were simulated based on a forward-time population simulator, which accounts for random mating, genetic drift, recombination and population growth rate, in genomeSIMLA [15]. We randomly selected 50 genes as a pathway for the simulations. A total of 1,038 SNPs within 20 KB to the genes in the pathway were selected. Three SNPs (*X_1_*, *X_2_* and *X_3_*) with minor allele frequencies 0.25, 0.15 and 0.15, each in different genes, were used as disease loci. A penetrance function similar to the one used in [16] was used to simulate the affection status:
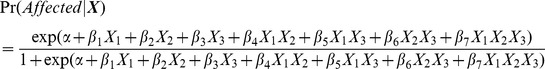
(1)where ***X*** is a vector of *X_1_*, *X_2_*, and *X_3_*, *α* is the parameter based on the disease prevalence, *β_1_*, *β_2_*, and *β_3_* correspond to the conditional marginal effects for *X_1_*, *X_2_*, and *X_3_*, *β_4_*, *β_5_* and *β_6_* correspond to the conditional interaction effects for the second-order interactions, and *β_7_* models the conditional interaction effects for the third-order interaction. *X_i_* is equal to 1 in the presence of at least one of the minor alleles at the locus *i* and equal to 0 if no minor allele is present. The disease prevalence was assumed to be 1%. We simulated 1,000 cases and 1,000 controls in each replicate of the simulations. We refer to the settings of these parameters (i.e. the number of genes in the pathway, the number of disease loci, the disease prevalence, and the number of cases and controls) as *Scenario 1*.

In addition to *Scenario 1*, we also changed the parameters one at a time for a more comprehensive simulation study. For *Scenario 2*, we simulated 500 cases and 500 controls. For Scenario 3, we changed the disease prevalence to be 5%. For Scenario 4, we simulated a larger pathway with 100 genes. A total of 1,527 SNPs within 20 KB to the genes in the pathway were used. We simulated an additional disease locus with a minor allele frequency of 0.25 for the large pathway. The disease locus has only main effects on the disease and the model for the other three disease loci is the same as *eq* (1).

We downloaded the Random Jungle package [17], which efficiently implements the RF algorithm, for the RF analysis in steps 2 and 3 in the TRF-pathway algorithm. We specified *K* as 2000 in all of our simulations as well as in the real data analyses. The default bootstrap procedure in the RF algorithm was used to determine the relative proportions of the samples in the training and test sets. To evaluate the type I error rates for the TRF-pathway, the parameters (*β_1_–β_7_*) were all specified as 0. A total of 5,000 replicates of simulated datasets were used to calculate the type I error rates. For power simulations, we first simulated a model (*Model 1*) with main effects only. The parameters *β_1_*, *β_2_*, and *β_3_* were specified as 0.92 and *β_4_*–*β_7_* were specified as 0 in *Model 1*. Then we simulated a multiplicative model similar to the model used in Chatterjee et al. [16]. That is, *β_1_*, *β_2_*, and *β_3_* were specified as *ϕ*, *β_4_*, *β_5_*, and *β_6_* were specified as 2*ϕ,* and *β_7_* was specified as 3*ϕ*. Therefore, the joint effect of two or three markers was the product of the main effects of the individual markers. *Model 2*, *Model 3*, and *Model 4* were simulated with *ϕ* equal to 0.18, 0.22, and 0.26, respectively. A total of 500 replicates of simulated datasets were used to calculate the power for each model.

We compared the power of the TRF-pathway with the RF-pathway and the set-based test in PLINK [18]. All of the 1,038 SNPs were provided as a set in PLINK. TagSNPs selected based on the LD measure *r^2^* of 0.5 were tested for association using a standard chi-square test. The mean of the chi-square statistics for SNPs with p-values <0.05, which is the default setting in PLINK, was used as the statistic for the pathway in PLINK. A permutation procedure is used to create a null distribution for the statistic and estimate the p-value. Therefore, the set-based test in PLINK does not need background genes across the genome for the statistic. In the following text, we refer to PLINK as the set-based test implemented in PLINK. In all of the simulation models, we specified the threshold as 1.64 for the variable importance scores, which corresponds to p-value of 0.05 in a one-tailed Z-test, in step 3 in the TRF-pathway algorithm.

## Results

### Simulations

**Figure 1:**
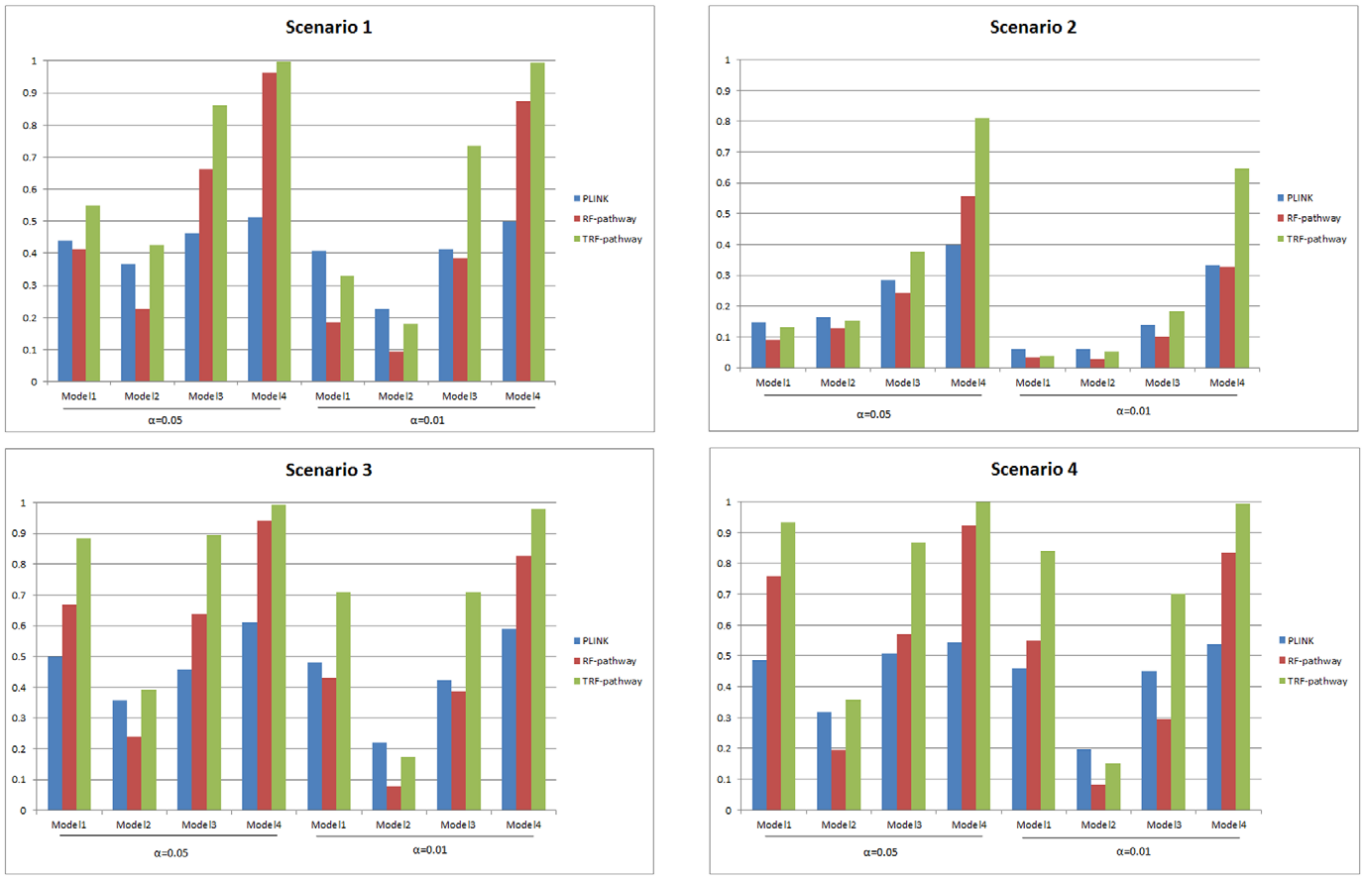
Power comparison of the TRF-pathway with PLINK and RF-pathway at the 0.05 and 0.01 significance levels.

The type I error rates for the TRF pathway, RF-pathway and PLINK at the 0.05 and 0.01 levels under different scenarios are shown in Table 1. As shown in Table 1, the simulation results suggested that the TRF-pathway and the RF-pathway both have the correct type I error rates close to the 0.05 and 0.01 nominal levels when *β_1_–β_7_* were all specified as 0. The power comparisons for the TRF-pathway with the RF-pathway and PLINK under the 4 scenarios were shown in Figure 1 at the significance levels of 0.05 and 0.01. In Figure 1 we can see that the TRF-pathway consistently has more power than the RF-pathway for all models. We can also see that PLINK can have more power than the TRF-pathway in *Model 1* and *Model 2*. PLINK can also have more power than the RF-pathway in *Models* 1, 2, and 3 across the 4 scenarios. However, with the increased multiplicative effects of gene-gene interactions in *Model 3* and *Model 4*, the TRF-pathway has significantly more power than PLINK. The results demonstrate that by using a two-stage approach, the TRF-pathway can improve power significantly when compared to the traditional RF-pathway approach. Moreover, the RF-based pathway methods can have more power than methods considering only main effects in the presence of strong gene-gene interaction effects.

**Table 1:** Type I error rates for the TRF-pathway, RF-pathway, and PLINK set-based tests.

	TRF-pathway		RF-pathway		PLINK	
	0.05	0.01	0.05	0.01	0.05	0.01
Scenario 1	0.048	0.012	0.049	0.009	0.052	0.012
Scenario 2	0.054	0.011	0.049	0.010	0.053	0.011
Scenario 3	0.058	0.011	0.047	0.010	0.047	0.009
Scenario 4	0.049	0.009	0.051	0.008	0.050	0.010

### A breast cancer GWAS analysis

We applied the TRF-pathway to a breast cancer GWAS dataset available at dbGaP [19], [20]. The dataset consists of 1,145 cases and 1,142 controls and 499,206 markers across the genome genotyped on the Illumina 550K platform. All the samples are Caucasian women. SNPs that are within 20 KB to a gene were assigned to the gene. We downloaded pathway definitions from the Kyoto Encyclopedia of Genes and Genomes (KEGG) pathway database for humans [21]. There were 208 pathway definitions used for the analysis.

Since Random Jungle assumes that there are no missing genotypes in the data, we imputed the missing genotypes in the sample using fastPHASE [22]. Genotypes with the highest likelihood were used to replace the missing genotypes. A total of 2,000 permutations were used to estimate the p-value for each pathway in the TRF-pathway algorithm.

Table 2 shows the pathways with p-values <0.01 identified by the TRF-pathway for the breast cancer GWAS. We also showed the number of genes and the number of SNPs used in step 3 in the TRF-pathway algorithm for each pathway in Table 2. None of the pathways in Table 2 can pass the Bonferroni threshold for multiple testing correction. However, interestingly, aminoacyl tRNA synthetases (AARSs) that are involved in the “Aminoacyl-tRNA biosynthesis” pathway in Table 2 have been shown to have implications for the etiology of breast cancer [23]. AARSs are essential for protein synthesis, and function as regulators and signaling molecules in biological processes [24]. One of the AARSs, lysyl-tRNA synthetase (KRS), was found to be over-expressed in the tumor regions of breast cancer patients [24].

**Table 2:** Pathway analysis results for the breast cancer GWAS data.

Pathway	No. Genes^1^	No. SNPs^2^	TRF P-value^3^	RF P-value^4^	PLINK P-value^5^
T cell receptor signaling pathway (hsa04660)	97	105	0.001	0.168	0.035
Maturity onset diabetes of the young (hsa04950)	25	27	0.003	0.043	0.048
Prostate cancer (hsa05215)	82	90	0.004	0.143	0.012
Aminoacyl-tRNA biosynthesis (hsa00970)	39	56	0.009	0.016	0.252

1 Number of genes in the pathway.

2 Number of SNPs used in the step 3 in the TRF-pathway algorithm.

3 P-values for the TRF-Pathway.

4 P-values for RF-Pathway.

5 P-values for PLINK set-based tests.

### A lung cancer GWAS analysis

We also applied the TRF-pathway to a lung cancer GWAS dataset from the Cancer Prevention Study II Nutrition Cohort (CPS-II) [25] available at dbGaP [20]. After QC, the dataset consists of 663 cases and 642 controls and 496,761 markers genotyped on the Illumina 550K platform. The subjects were collected by the American Cancer Society between 1992 and 2001 across all U.S. states. The same procedures as the breast cancer analysis were used to impute missing genotypes. The same pathway definitions from KEGG were used for the analysis.

Table 3 shows the pathways with p-values <0.01 identified by the TRF-pathway for the lung cancer GWAS. Similar to Table 2, we showed the number of genes and the number of SNPs used in step 3 in the TRF-pathway algorithm for each pathway in Table 3. Interestingly, the TRF-pathway identified the p53 signaling pathway, which is associated with many human cancers, with p-value 0.006. The MDM2 gene, which is a key negative regulator of p53 activity, is a candidate gene for non-small cell lung cancer [26]. The p53 and MDM2 genes have also been shown to interact with smoking for lung cancer in a Chinese population [27].

**Table 3:** Pathway analysis results for the lung cancer GWAS data.

Pathway	No. Genes^1^	No. SNPs^2^	TRF P-value^3^	RF P-value^4^	PLINK P-value^5^
Cyanoamino acid metabolism (hsa00460)	7	19	0.001	0.092	0.192
Fc gamma R-mediated phagocytosis (hsa04666)	88	133	0.002	0.010	0.381
p53 signaling pathway (hsa04115)	66	50	0.006	0.064	0.208
Pentose phosphate pathway (hsa00030)	22	25	0.008	0.208	0.506

1 Number of genes in the pathway.

2 Number of SNPs used in the step 3 in the TRF-pathway algorithm.

3 P-values for the TRF-Pathway.

4 P-values for the RF-Pathway.

5 P-values for PLINK set-based tests.

## Discussion

We developed the TRF-pathway, which is a powerful two-stage RF-based pathway analysis method extended from the RF-pathway. Unlike many pathway analysis methods that consider only main effects of genes, the TRF-pathway considers both main effects of genes and gene-gene interactions. We used simulations to verify that both the TRF-pathway and the RF-pathway are valid tests for pathway association under the null hypothesis that none of the SNPs within a pathway are associated with the disease. We then used simulations to demonstrate that by employing a two-stage design, statistical power can be significantly increased in the TRF-pathway compared to the RF-pathway.

Our power comparisons suggested that when there are only main effects or the effects of gene-gene interactions are not strong (i.e. *Model 1* and *Model 2*), PLINK can have more power than the TRF-pathway and RF-pathway. This is not surprising as PLINK tests specifically for main effects. However, when the effects of gene-gene interaction are strong, the TRF-pathway has significantly more power than PLINK. Therefore, in practice, the TRF-pathway should be used as a tool that is complementary to the methods considering only main effects such as PLINK.

The score *R*, which is the prediction rate based on the SNPs with importance scores greater than a threshold in a pathway, should not be used as an unbiased prediction error rate for the SNPs due to the selection bias of the SNPs in step 3 in the TRF-pathway algorithm. We did not calculate the unbiased prediction error rate for the SNPs in the TRF-pathway algorithm because the purpose of the TRF-pathway is to test the association of SNPs in the pathway. N-fold cross-validation technique can be used to estimate the unbiased error rate for the important SNPs in step 3. For example, based on a 10-fold cross-validation procedure, each 9/10 of the samples can be used in the first stage of the TRF-pathway algorithm as a training set to find the significant SNPs. The remaining 1/10 of the samples can be used as a test set to calculate the score *R* for the significant SNPs in step 4. Then the prediction error rate is the average of *R* over 10 replicates. However, sample size will be reduced due to the partition of the data. Our simulation suggested that this resulted in a significant loss of power (Data not shown). Alternatively, an independent dataset can be used to calculate the unbiased prediction error rate for the significant SNPs in step 3 in the algorithm.

The significant results shown in Tables 2 and 3 for the breast cancer and lung cancer GWAS analyses did not pass the Bonferroni threshold for multiple testing correction. However, tests for pathways are not independent because pathways can share common genes. Therefore, the Bonferroni correction can be conservative. Moreover, an interesting pathway (hsa00970) identified by the TRF-pathway has been shown to have implications for breast cancer etiology. The p53 pathway (hsa04115), which contains candidate genes for lung cancer, was also identified by the TRF-pathway. This demonstrates that the TRF-pathway can be a powerful tool for identifying candidate pathways associated with diseases.

Unlike some pathway methods that calculate gene-specific scores for pathway statistics [1], [6], the TRF-pathway uses all SNPs within genes in a pathway for the joint inference without considering gene-specific information such as gene sizes or groupings of SNPs within genes. Power studies suggested that pathway methods without calculating gene-specific statistics such as PLINK can still be more powerful than methods that specifically calculate gene scores [6]. However, it will be of interest to know how gene-specific information can improve power for the TRF-pathway. We are investigating how to incorporate gene-specific information such as gene sizes and LD structures in the RF model for pathway analysis.

In summary, we used simulations and applications to breast cancer and lung cancer GWAS datasets to demonstrate that the TRF-pathway is a powerful pathway analysis tool. The TRF-pathway is implemented in a PERL script. The script uses the PLINK software to generate input files for Random Jungle (with –recodeA option) and uses Random Jungle for the RF calculations. A more efficient program of the TRF-pathway using C++ will be implemented so that the TRF-pathway can be applied to a large set of pathways. The script is freely available at http://sourceforge.net/projects/trfpathway/.
